# The Combination of Physical Exercise with Muscle-Directed Antioxidants to Counteract Sarcopenia: A Biomedical Rationale for Pleiotropic Treatment with Creatine and Coenzyme Q10

**DOI:** 10.1155/2017/7083049

**Published:** 2017-09-20

**Authors:** Michele Guescini, Luca Tiano, Maria Luisa Genova, Emanuela Polidori, Sonia Silvestri, Patrik Orlando, Carmela Fimognari, Cinzia Calcabrini, Vilberto Stocchi, Piero Sestili

**Affiliations:** ^1^Department of Biomolecular Sciences, University of Urbino Carlo Bo, Urbino, Italy; ^2^Department of Life and Environmental Sciences (DISVA), Marche Polytechnic University, Ancona, Italy; ^3^Department of Biomedical and Neuromotor Sciences, Alma Mater Studiorum, University of Bologna, Bologna, Italy; ^4^Department for Life Quality Studies, Alma Mater Studiorum, University of Bologna, Rimini, Italy

## Abstract

Sarcopenia represents an increasing public health risk due to the rapid aging of the world's population. It is characterized by both low muscle mass and function and is associated with mobility disorders, increased risk of falls and fractures, loss of independence, disabilities, and increased risk of death. Despite the urgency of the problem, the development of treatments for sarcopenia has lagged. Increased reactive oxygen species (ROS) production and decreased antioxidant (AO) defences seem to be important factors contributing to muscle impairment. Studies have been conducted to verify whether physical exercise and/or AOs could prevent and/or delay sarcopenia through a normalization of the etiologically relevant ROS imbalance. Despite the strong rationale, the results obtained were contradictory, particularly with regard to the effects of the tested AOs. A possible explanation might be that not all the agents included in the general heading of “AOs” could fulfill the requisites to counteract the complex series of events causing/accelerating sarcopenia: the combination of the muscle-directed antioxidants creatine and coenzyme Q10 with physical exercise as a biomedical rationale for pleiotropic prevention and/or treatment of sarcopenia is discussed.

## 1. Introduction

Sarcopenia, that is, age-associated muscle weakness and reduced muscle mass, is characterized by a decrease in muscle fiber number and size, slower contraction speed, shift in fiber type composition, and changes in various metabolic parameters. These features link to muscular dysfunction [1], namely, the loss of strength and performance, with a risk of adverse outcomes such as falls and fractures, impaired ability to perform activities of daily living, and increased risk of death [2]. Sarcopenia is not a disease but a syndrome caused by multiple factors [3]: among these, oxidative stress and mitochondrial dysfunction have long been reported. Although the oxidative stress theory of aging is under continuous reappraisal [4] taking into account, for example, the hormetic nature of reactive oxygen species (ROS) along with the merging of different theories in a new one, there is accumulating evidence that the biological process of aging is characterized by oxidative stress and mitochondrial dysfunction [5], which in turn result in a significant decline of aerobic capacity in the senescent muscle [6].

Although the reasons for the increased ROS production in aged subjects are still not entirely clear, the analysis of the literature shows that age-related defects in the mitochondrial respiratory chain (MRC) are considered a crucial factor [7]. In particular, the production of superoxide by complex I (NADH-ubiquinone oxidoreductase) is strongly dependent on the plasticity of the supramolecular organization of the MRC [8], which may modulate the conformational state of this multisubunit enzyme complex, the stability of its ROS-generating domain, and the consequent leak of electrons [9], despite the absence of manifested genetic defects. Furthermore, the arrangement of active sites within the supercomplex architecture may help to limit ROS production by complex III (ubiquinol-cytochrome c oxidoreductase) [10]. In our hypothesis, the dissociation of respiratory supercomplexes may therefore link to age-associated oxidative stress and energy failure in a vicious circle also including membrane lipid peroxidation and mtDNA damage as possible concurring factors [11].

Such a situation may lead to a profound imbalance of ROS physiological signalling [12], which plays a pivotal and positive role in muscle homeostasis and function, particularly in the adaptive response to physical exercise [13]. In fact, a relatively mild action of ROS produced during exercise leads to ergogenic and adaptive stimuli. Otherwise, if the cell is overwhelmed by the action of ROS, then subcellular damage, signalling dysregulation, and aging will take place.

The dual nature of ROS (i.e., as a general feature of detrimental damage to cellular structures in aging and as secondary messengers) is also highlighted by recent results [14] illustrating that increasing ROS production specifically from over-reduced coenzyme Q (CoQ) pool and through respiratory complex I reverse electron transport generates a ROS signal necessary for cell homeostasis, which extends Drosophila lifespan despite reducing mitochondrial respiration. Scialo et al. [14] conclude that, if such mechanism is conserved in mammals, manipulation of the redox state of CoQ may be a strategy for the extension of both mean and maximum lifespan and the road to new therapeutic interventions for aging and age-related diseases.

The age-related imbalance between ROS and antioxidant (AO) defences is also reputed as a primary cause of detrimental chronic inflammatory reactions in human skeletal muscle [15, 16]. Another emerging line of reasoning considers the contribution of epigenetic pathways both at nuclear and at mitochondrial levels. The epigenetic mechanisms involved in the aging process include alterations of the DNA methylation status, modifications of the histone tails (mainly acetylation and methylation), and changes in the expression of noncoding RNAs [17]. These modifications are strictly dependent on the cellular energy status and can be influenced by nutritional habits and cellular stress conditions, including excessive mitochondrial ROS levels [18].

There is currently no optimal treatment for sarcopenia. Potential intervention strategies for relieving the symptoms of the syndrome include physical activity, hormone administration, caloric restriction, and nutritional interventions including intake of supplementary AOs [19].

As to physical activity, it is increasingly considered as a promising strategy to limit age-related sarcopenia due to the potent associated adaptive response [20]. In fact, exercise interventions improve the quality of life in older adults and, in particular, resistance exercise effectively modulates muscle mass and function [21, 22]. From a molecular point of view, for example, exercise training has been shown to ameliorate systemic AO capacity [23] and to delay the age-related decrease of DNA repair capacity [24] and of mitochondrial biogenesis [25].

At the same time, since increased muscle contraction in elderly people may lead to unmatched ROS production, it has been suggested to adopt supplementation regimens with dietary AOs. However, this assumption is controversial due to the lack of strong evidence indicating adverse and/or positive effects of combining exercise training with dietary supplementation with generic AOs [26].

Considering this last notion, that is, the hormetic nature of ROS and the complexity of the diverging responses they elicit, the combination of exercise training with rationally selected, muscle-directed AOs, namely, creatine (Cr) and coenzyme Q10 (CoQ10), is discussed in this review.

## 2. The Multifactorial Link between Sarcopenia and Oxidative Stress

Sarcopenia has been defined as the presence of both low muscle mass and low muscle function that occurs with advancing age [1]. Age-associated muscle weakness and reduced muscle mass are characterized by a decrease in muscle fiber number and size, slower contraction speed, shift in fiber type composition, and changes in various metabolic parameters. These alterations are associated with an age-related decline in several whole-body physiological variables, such as maximal oxygen uptake, endurance performance, insulin sensitivity, muscle power, and strength [27–29].

Here, we focus on the relationships between age-associated oxidative imbalance/stress and sarcopenia. Since oxidative stress is not the only cause of muscle aging, for a more comprehensive review on the etiopathological factors and mechanisms involved in sarcopenia, see [30].

Sarcopenia is caused by both intrinsic and extrinsic factors [3]. Among these factors [30], oxidative stress and mitochondrial dysfunction are known to participate in the aging process of the skeletal muscle and other organs [31, 32]. Muscle mitochondrial dysfunction may cause both loss of mitochondrial density and impairment of oxidative phosphorylation due to lower respiratory capacity resulting in reduced ATP production [6]: this evidence led to the proposal of the “mitochondrial theory of sarcopenia” [33].

The age-associated increase of ROS causes damage to macromolecules, in particular, to MRC components, which further increases the production of free-radicals resulting in the accumulation of mitochondrial damage and ineffective mitochondria quality control [31, 33].

Defective operation of the enzyme complexes of the MRC constitutes a key mechanism involved in the age-associated loss of bioenergetic reserve capacity and enhanced electron leakage from complexes I, II, and III. However, it is worth noting that the primary event responsible for aging may be the structural damage of both lipids and proteins, as induced by ROS directly and indirectly via the alteration of the signalling pathways modulating the mitochondrial activity [11]. Peroxidation of bioactive lipids and phospholipids of the inner mitochondrial membrane can perpetuate a vicious circle by hampering the supramolecular organization of the respiratory supercomplex I_1_III_2_ (SC) [34] thus favouring further generation of the superoxide anion radical by complex I, which eventually leads to catastrophic metabolic deficiency of mitochondria. One of the first signals in mitochondria is oxidized cardiolipin (CL) [35]. Bound CL molecules are seen in the crystal structures of the respiratory complexes and have been recently shown to stabilize, indeed, the respiratory supercomplexes. Selective peroxidation of CL, which in the animal variant carries polyunsaturated fatty acid chains, was demonstrated in response to mitochondrial oxidative stress in various experimental models, and among others, a consequence of CL peroxidation is the alteration of the supercomplex assembly with impaired MRC function; see [36] for review. Indeed, disaggregation of SC and the consequent loss of CoQ buried in its interface still allow electron transfer through a free CoQ pool in the membrane lipid bilayer, but in a less efficient way because the CoQ content in mitochondria is not saturating for maximal respiratory activity. In fact, Lenaz et al. [36] demonstrated that electron transfer between complex I and complex III (i.e., NADH-cytochrome c oxidoreductase activity) in isolated bovine heart mitochondria can take place at a high rate as long as the SC organization is not destroyed by *in vitro* treatment with high-dose n-dodecyl-*β*-D-maltoside. These data can be interpreted as maintenance of facilitated CoQ channeling within the respiratory SC as long as the SC itself is preserved, whereas NADH-cytochrome c oxidoreductase activity significantly decreases when electron transfer in the CoQ region occurs under conditions of less efficient collision-based pool behaviour (see also section 6 ). As a consequence, the alteration of electron transfer may elicit strong enhancement of ROS generation. Several additional observations in cellular and animal models link together SC dissociation and enhanced ROS production [37–39].

Muscle cell aging is also characterized by a build-up of oxidatively modified proteins. The steady-state level of oxidized proteins depends on the balance between the rate of protein oxidative damage and the rates of protein degradation and repair. The proteasomal system is the major intracellular proteolytic pathway implicated in the degradation of oxidized proteins, and its function declines progressively with age. Therefore, the accumulation of oxidized proteins can be due to increased protein damage, decreased oxidized protein degradation and repair, or the combination of both mechanisms [40].

The complex scenario described so far also implies derangement in ROS-mediated cell signalling [41]. Redox signalling refers to a unique signal transduction pattern wherein some ROS serve as signalling molecules to modulate specific residues of the targets that cause changes in enzyme activity, transcription factor/cofactor association/dissociation, DNA binding, and gene expression [42, 43].

Alteration of the signalling network involving ROS has received increasing recognition over the past two decades [12]. Such alteration/dysregulation phenomena caused by excessive ROS also play an important role in sarcopenia. To this regard, however, it is important drawing some considerations. Firstly, ROS are not only detrimental: indeed, physiological ROS levels promote the activation of signalling cascades contributing to muscle homeostasis; secondly, the concept of “excessive” is not absolute, depending on the balance with cellular AO defences and cellular conditions [44]; and thirdly, even the lack of ROS may cause a detrimental cessation of signalling cascades important for muscle physiology [5, 44]. Given this premise, the most relevant redox-sensitive signalling pathways which may be imbalanced by excessive ROS are NF-*κ*B [45], MAPKs [46] and PGC-1*α* [47], and Akt/mTOR [48]. Indeed, all these paths are activated by exercise and one of the major mechanisms of their activation relies on the increase of H_2_O_2_ levels [5] within a physiologically compatible range.

For example, NF-*κ*B responsive elements are present in the promoter regions of genes encoding catalase, glutathione peroxidases, and Mn- and Cu-Zn-superoxide dismutase [49]. PGC-1*α* is an inducible transcriptional coactivator participating in almost all aspects of mitochondrial functions ranging from energy fuel selection, muscle fiber differentiation and transformation, AO gene expression, and mitochondrial biogenesis to fusion and fission dynamics [50, 51]. PGC-1*α* has been implicated as a major regulator of the mitochondrial biogenesis through the interaction with NRF-1 that stimulates the transcription of many mitochondrial genes as well as TFAM, a direct regulator of mitochondrial DNA replication and transcription [52]. PGC-1*α* has a direct role in preserving muscle plasticity [47], and its age-related downregulation may play an important role in the decline of mitochondrial biogenesis and turnover contributing to the aetiology of sarcopenia [53, 54].

Mitochondrial function also depends on the coordination of nuclear and mitochondrial genomes, and therefore, the transcription and translation of both are coregulated. In fact, on one hand, the nucleus controls the mitochondrial function by promoting biogenesis and regulating mitochondrial activity to meet the cellular needs (“anterograde-regulation”); on the other, mitochondria can control the expression of nuclear genes modifying cellular function by reprogramming its metabolism (“retrograde response”). The study of mitonuclear communication has received great interest since it constitutes a robust network maintaining cellular homeostasis, regulating the adaptation to a variety of stressors, and promoting longevity [18].

With regard to mTOR signalling, its interplay with ROS is very complex, since ROS play both activating and inhibitory roles. Increasing evidence suggests that mTORC1 is a critical ROS mediator [55]. Recently, the effect of excessive ROS accumulation on the PI3K/AKT/mTOR signalling axis was evidenced by the near complete inhibition of mTORC1 activity as reflected by a decrease of the phosphorylated forms of proteins that are immediately downstream of the mTORC1 complex, namely, 4EBP1, p70S6K, and rpS6 [56, 57].

It is worth noting that the events described in this section are not limited to free radicals participating in signalling cascades themselves but may also be related to changes in the redox potential of different cellular compartments involved in redox-sensitive cascades according to their intrinsic susceptibility [58].

Oxidative stress does not only act as a direct effector of inflammation but also appears to be a primary causal factor in producing a chronic state of low-grade inflammation through activation of redox-sensitive transcription factor NF-*κ*B [59]. Age-associated mitochondrial overproduction of ROS not only deregulates intracellular signalling but also affects intercellular communication. In particular, oxidative damage promotes senescence of cells, which in turn acquire a senescence-associated secretory phenotype characterized by the release of proinflammatory cytokines and several miRNAs [60] that alter the intercellular milieu and finally lead to inflammaging. Moreover, low-grade inflammation alters cellular protein metabolism to favour proteolysis over synthesis, thereby accelerating muscle atrophy [61] probably through the accumulation of TNF*α* and proinflammatory cytokines that in turn leads to protein degradation via proteasome activation and reduced skeletal muscle protein synthesis.

Recent studies support the idea that aging impacts on muscle stem cell function in terms of their capacity to self-renew, thus altering the composition of muscle niche. Moreover, a growing body of evidences points towards crosstalk between intrinsic (ROS, mitochondrial dysfunction, etc.) and extrinsic (circulatory factors and altered muscle niche) factors that on the whole contribute to poor efficiency of the muscle repair capacity at a geriatric age [3]. Muscle niche is regulated by growth and trophic factors, cytokines, and extracellular miRNAs carried by exosomes [62]. Extracellular miRNAs are modulated by exercise, immobilization, and muscular diseases. In addition, the expression of several miRNAs is also altered during aging [63].

Counteracting muscle mass loss during aging is a strong predictor for longevity in humans [64]. However, there is currently no optimal treatment for sarcopenia: potential intervention strategies include, among others, physical activity and nutritional supplementation (e.g., AOs) [19].

## 3. The Hormetic Nature of Exercise Training in the Elderly

Physical exercise has been shown to activate various redox-sensitive signalling pathways that control mitochondrial biogenesis, AO defence, inflammation, protein turnover, apoptosis, and autophagy [5, 65]. Although these stimulatory effects of exercise decline with aging, they are not completely abolished. As a consequence, the stimulation of these residual capacities through exercise training represents an effective countermeasure against aging-associated transcriptional remodelling. In line with this evidence, it has been shown that the magnitude of increase in aerobic capacity following endurance training in older individuals is similar to that in younger subjects [66]. Thus, aged people can still benefit from regular physical activity in the appropriate forms and at proper intensity to preserve muscle function.

Indeed, there is a growing body of evidence showing that exercise interventions improve the quality of life in older adults [21] and particularly resistance exercise effectively modulates muscle mass and function in elderly people [22, 67].

Exercise training impacts muscle function through epigenetic mechanisms including histone deacetylation and loss of promoter methylation that modify exercise-responsive gene expression (i.e., PGC-1*α*, TFAM, and MEF2A), even after an acute bout of exercise, triggering structural and metabolic adaptations in skeletal muscle [68].

Physical exercise-mediated muscle health and longevity also involve sirtuin-1-regulated pathways promoting mitochondrial function and reduces the production of ROS through regulation of PGC-1*α*, the master controller of mitochondrial biogenesis [69], along with increased NRF-1 and TFAM levels. This results in increased mitochondrial oxidative capacity and ATP production, enhanced expression of tricarboxylic acid cycle and MRC enzymes, higher fatty acid oxidation, and mitochondrial morphological changes [47].

In particular, increased expression of PGC-1*α* in muscle not only promotes mitochondrial biogenesis, enhances aerobic metabolism, and mimics the benefits of endurance training but also augments AO defences [53]. In addition, increased muscle PGC-1*α* activity delays several age-related metabolic defects, such as chronic inflammation and reduction in insulin sensitivity, thus causing important consequences at systemic level. These effects of PGC-1*α* activity in muscles presumably result from several PGC-1*α*-regulated processes, including resistance to oxidative stress [53], inhibition of atrophy [70], regulation of muscle metabolism, and release of myokines [71].

Moreover, using animal models, it has been demonstrated that endurance exercise rescues mitochondrial defects and premature aging of mice with defective proofreading exonuclease activity of mitochondrial DNA polymerase *γ* [72]; exercise protects animals from neurodegeneration [73, 74] and can extend lifespan in rats [75], and it has been hypothesized that it could improve life expectancy in humans [76].

In older humans, beyond muscle mass, resistance training could modify the balance between oxidants/AOs by improving AO defences. According to this view, it has been reported that resistance training reduces the 8-OHdG/creatinine ratio in urine, an effect that Parise et al. [77] hypothesized to depend on an upregulation of glutathione peroxidase activity [77]. However, recent literature suggests that improvements are closely related to the intensity of training protocols that must contain sufficient volume for each muscle group (3–5 sets, 10 repetitions) and intensities between 50 and 80% of 1 repetition maximum [41].

Increased muscle loading induces adaptive responses through a myriad of intracellular signalling events such as activation of the Akt/mTOR [48, 78, 79] and myostatin pathways [80]. In addition to myostatin, contracting muscle produces other myokines including insulin-like growth factor-1 (IGF-1) [81]. Although muscle-derived IGF-1 is not detected in the circulation [82], it induces muscle hypertrophy in an autocrine/paracrine fashion following exercise [83].

Besides stimulating muscular anabolism, exercise inhibits protein degradation. Indeed, PI-3K/Akt inhibits the forkhead box transcription factor O (Fox-O), a potent inducer of the ubiquitin-proteasome system, and mTOR decreases caspases activity. Furthermore, physical inactivity stimulates Fox-O, which can also inhibit the mTOR pathway [84]. Furthermore, recent evidence shows that physical exercise, in the form of both endurance and resistance training, induces autophagy with metabolic beneficial effects [85, 86]. Although the function of exercise-induced autophagy is still unclear, it is widely accepted that autophagy may be particularly important in a sarcopenic muscle by promoting the turnover of cellular components through the removal of damaged proteins and organelles.

Since satellite cells are critical players in skeletal muscle plasticity and repair, they have been suggested to be involved in the development of sarcopenia. Even if there is controversy as to whether satellite cells actually decrease in number with aging [87–89], their proliferative response and regenerative capacity are reduced in the aged muscle [90, 91]. Evidence indicates that reduced regenerative function is more likely the result of a less optimal cellular environment than the cells being deficient due to age [3]. Exercise can enhance the activity of stem cells, including muscle satellite cells. Recent studies show that the increase in muscle mass and myonuclear content in response to exercise is accompanied by an increase in the number and activation status of satellite cells [92]. On the whole, it has been shown that regular exercise has a significant effect on the prevention of these age-associated losses [93]. Chronic exercise could be considered as an effective measure against age-induced oxidative stress by improving pleiotropic responses able to scavenge ROS and repair damaged molecules/organelles [94].

Nevertheless, the available information shows that acute exercise increases ROS production and oxidative stress damage in older adults; this paradoxical effect is due to the ability of the exercise itself to increase the formation of ROS to a level that may induce significant yet tolerable damage, which can, in turn, promote beneficial adaptations [95].

In general, exercise at high intensity or for a long duration can enhance ROS and free radical production, induce damage in various tissues [96], and decrease circulating AOs (uric acid, SH-groups, alpha-tocopherol, beta-carotene, and retinol) [97]. However, exercise at moderate intensity and duration can generally be regarded as an upregulator of AO defences and a downregulator of ROS/free radical production under basal conditions and during exercise [98]. A recent study by Bouzid et al. [41] compared the effects of regular physical activity at high and moderate intensities on oxidative stress in older adults. The same authors concluded that both low and high physical exercise levels help to maintain better AO defences, namely, SOD, glutathione peroxidase, and glutathione reductase in older adults.

## 4. Nutritional Antioxidant Intervention in the Elderly Subjected to Physical Exercise

The intake of supplemental AOs in training elderly people should in principle abrogate the ROS-related negative effects elicited by muscle contraction without affecting the positive ones.

Despite this rationale, the effects of supplementation with AOs in training elderly people on physical performance and muscle conditions are controversial: for example, some studies using resveratrol, vitamin C, and vitamin E showed protective actions resulting in the reduction of muscle damage [99–101], and supplementation with an AO mixture (vitamins E and A, rutin, zinc, and selenium) plus leucine induced an anabolic response in an old muscle [102]. Conversely, other preclinical trials using vitamins C and E demonstrated that AO supplementation may abrogate the benefits of exercise, probably by silencing ROS signalling [103]. Ristow et al. [104] and Gomez-Cabrera et al. [105] have recently reported that AO supplementation can decrease training efficiency and prevent exercise-induced mitochondrial biogenesis in healthy humans. Furthermore, a recent study showed no effects of vitamins C and E supplementation on muscle function in response to exercise-induced adaptation [106].

Another study dealt with N-acetyl-cysteine administration aimed at fostering reduced glutathione availability during an 8-day period after eccentric exercise-induced muscle damage. In this study, it has been shown that, although redox status alterations attenuate oxidative damage and inflammation, they may delay muscle long-term recovery by interfering with intracellular signalling pathways [107].

Moreover, protein supplementation (e.g., whey and soy proteins and isoflavone-enriched soy proteins) has been proposed as a new AO strategy primarily because of its capacity to enhance the availability of reduced glutathione and the activity of the corresponding AO enzyme system. However, there is still a lack of information about the anabolic potential of dietary protein intake and protein supplementation in elderly people with increased systemic inflammation [108].

On the whole, AO supplements may optimize the training effects by protecting against exercise-induced ROS overproduction, but on the other hand, overdosed supplementation may blunt training beneficial adaptive effects.

However, it is important to note that these studies have been conducted using mainly vitamins C and E and results have been generally extended to the broader category of AOs. This term represents a rough simplification that can be misleading, since individual AOs differ in their mechanisms of action, redox affinity, bioavailability, tissue kinetic, intracellular distribution, dose dependence, and additional properties. Moreover, conclusions referring to a young and healthy human population should not be directly applied to the elderly population characterized by a significant impairment of redox balance underlying age-related muscle modifications and impaired adaptive response to exercise training.

In the same direction, Jackson competently and thoughtfully commented: “the combination of physiological and pathological roles of ROS imply that interventions based on a simple suppression of ROS activities through use of non-specific AOs are unlikely to retard or improve the age-related declines in muscle mass and function” [109]. Hence, the equation “more AOs – less muscle aging” is not an automatic truth and indeed, with respect to the prevention of sarcopenia, the question of whether supplementation with AOs is positive should be better addressed, with a particular focus on the possible broader biological properties of selected AOs.

## 5. Creatine, New Perspectives from the Past

Cr is among the most studied and long known molecules in modern and contemporary biology. Since its discovery, more than 150 years ago by Michel Chevreul, thousands of studies dissected and identified its central role in cellular energetics as the substrate for creatine kinases. According to its ergogenic function, the bulk of body Cr and Cr phosphate (CrP) is concentrated in skeletal muscles; body stores of Cr are maintained physiologically by nutritional intake through meat and fish consumption and endogenous biosynthesis [110]; and oral Cr supplementation further increases plasma Cr levels and intramuscular Cr/CrP content in young, adult [110], and elderly people [111].

Today, Cr has a strong reputation as a safe nutritional supplement promoting a number of health benefits not limited to sport practitioners [112]. This notion has been recently strengthened by the official Position of the International Society of Sports Nutrition [113], which also ruled out the concerns on its possible renal toxicity.

In particular, Cr, along with positively affecting muscle mass and performance [114–116], has been shown to favour myogenesis in normal and adverse conditions [117], to increase the expression level of specific muscle regulatory factors [118–121], to exert a trophic action on muscle cells [114], and to exert mild AO [122, 123] and anti-inflammatory [124, 125] activity. Thus, a new concept of a Cr biochemical and physiological role has emerged pointing to its multiple, not only ergogenic, effects [112, 116, 126]. In this light, Cr can be regarded as a nutritional supplement fulfilling a number of requisites which might be important in delaying the onset and the progression of sarcopenia [127, 128]. In this section, a summary of the multiple effects caused by Cr will be discussed.

The first evidence for an “AO-like” activity of Cr was reported by Matthews et al. [129] showing that oral Cr, in analogy with established AOs such as N-acetylcysteine, attenuated the hydroxyl radical and peroxynitrite generation in nitropropionic acid-intoxicated rats (an animal model of Huntington's disease) and prevented brain damages. The first report of the direct AO activity of Cr was provided by Lawler et al. [122] in an acellular setting (where Cr showed its ability to scavenge ABTS^+^, O_2_•^−^, and OONO^−^) and by Sestili et al. [123] in different mammalian cell lines challenged with a panel of oxidative stressors (H_2_O_2_, t-butyl-hydroperoxide, and peroxynitrite). In this study, intracellular, but not extracellular, Cr at nutritionally attainable concentrations was mildly but significantly cytoprotective towards the three toxic species [123].

Since then, many authors reported that Cr exerts direct and indirect AO effects in different *in vitro* and *in vivo* experimental settings/conditions in which oxidative stress takes place. For example, *in vitro* settings showed protection from the exposure to H_2_O_2_, t-butyl-hydroperoxide, and peroxinitrite [122, 123, 130], from oxidative damage to mtDNA [130–132] and RNA [133], from H_2_O_2_-induced arrest of myogenesis [117, 121], from UV rays damage [131, 132], and from glutamate-induced nitrosative and oxidative stress [134]. Animal studies showed that supplemental Cr protects from the toxicity of nitropropionate [129], of the mitochondrial electron transport inhibitor rotenone via direct AO activity [135], and of the proconvulsant drug pentylenetetrazole [136] and also demonstrated that Cr ameliorates the AO reservation against oxidative stress in exercise-trained ovariectomized hamsters [137], decreases ROS content with no changes in expression and activity of AO enzymes in rat skeletal muscle after 6 days supplementation [138], and reduces lipid peroxidation markers in exercising rats fed for 26 days with a Cr-enriched diet [139]. Human studies showed significantly lower accumulation of urinary 8-OHdG and plasma malondialdehyde in trained adults after 1 week of Cr supplementation [140, 141], limited short-term oxidative insults after the Wingate test (1 week Cr supplementation) [142], and reduced oxidative stress in a steady-state test at 75% VO_2_(max) after 5 days supplementation [143].

The mechanisms responsible for the AO activity of Cr are complex and have been extensively reviewed elsewhere [116, 144]. These mechanisms do include not only direct but also indirect interactions. With regard to the latter, for example, Cr induces peroxiredoxin-4 and thioredoxin-dependent peroxide reductases, two important AO enzymes located in the cytoplasm and mitochondria, respectively [145], and in addition, the same thioredoxin and peroxiredoxin system could benefit from the increased NADPH resynthesis due to the Cr-induced higher ATP availability [114, 116, 126, 146]; Cr has been shown to activate AMPK which in turn might promote cellular adaptive responses aimed at overcoming oxidative stress [117, 147, 148]; supplemental Cr may increase intracellular levels of arginine, which also can act as an AO [149]. Thus, the mild AO activity of Cr is likely to result from the superposition of multiple mechanisms.

Interestingly, the protective activity of Cr against acute oxidative stress, in spite of a scavenging potency lower than that of established AOs [122], proved to be better as compared to the latter in specific conditions [117, 121]. For instance, with regard to cultured muscle cell settings (C2C12 cells), a comparative study between the protective activity of Cr versus the reference AOs Trolox [150] or N-acetyl-cysteine [146] toward the cytotoxicity and the myogenic arrest caused by H_2_O_2_ showed that while Cr significantly attenuated both the toxic responses, the two reference AOs were not capable to prevent the latter. Such findings imply that in order to counteract specific adverse situations caused or exacerbated by oxidative stress, the “generic” capacity of acting as an AO might not be sufficient. Indeed, Cr, beyond acting as a mild AO, has other peculiar features which concur to muscle cell rescue as discussed below.

A prominent and qualifying feature of Cr is its particular tropism for skeletal muscle, where the bulk of body Cr is actively stored up to 30 mM [3]: this very high, tissue-specific concentration might selectively enhance its AO activity in the muscle compartment.

Furthermore, Cr shuttles energy from the site of production (mitochondria) to the site of consumption (cytoplasm) [116]. As a consequence, the particularly high gradient of Cr in mitochondria and across the mitochondrial membranes is likely to focus its activities in this critical subcellular compartment (see also above). Indeed, according to the mitochondrial theory of aging, a vicious circle (see section 1 ) entailing the overgeneration of ROS by the MRC and damage to both mitochondrial and cellular structures leads to defective mitochondrial respiration and further increase in mitochondrial ROS production and oxidative damage [109]. ROS damage to mitochondria deteriorates their functionality, integrity, and networking, which represent fundamental requisites for muscle differentiation [117, 151–161] whose imbalance is associated with muscle decline conditions such as sarcopenia [162]. As stated above and in line with this mitochondrially oriented activity, Cr increases the content of mitochondrial thioredoxin reductase, an important AO enzyme, in myotube cultures [145].

According to this peculiar mitochondrial tropism, we recently demonstrated that the addition of Cr to H_2_O_2_-injured differentiating C2C12 cells prevented the collapse of their mitochondria, attenuated the level of CL peroxidation, restored the respiration capacity, and prevented the fragmentation of the mitochondrial networks [117]. Cr was also found to increase mitochondrial mass and, accordingly, to stimulate the transcription level of PGC-1*α*, [163, 164], in both control and H_2_O_2_-injured differentiating C2C12 cells. Finally, in the same cells and conditions and according to previous reports [148], Cr per se caused an increased phosphorylation of AMPK which, along with the increased CrP and ATP levels found in supplemented cells, concurs to a better handling of cellular energy to overcome critical situations [117, 121].

With regard to this last concept, it is worth considering that the most authentic, unique feature differentiating Cr from generic AOs is obviously its role in cellular energetics. Cr improves cellular energy state (CrP/ATP ratio), facilitates intracellular energy transport through the CrP circuit, and enhances the overall CrP pool [116]. Increased CrP levels can be utilized by Cr kinase to transphosphorylate ADP to ATP for various ATP-dependent processes, including those required for muscle contraction [116]. In addition, better CrP/ATP ratios improve the cellular calcium handling and homeostasis and maintain an adequate MRC activity thus preventing ROS leakage [116]. Indeed, the ATP highly consuming calcium pump of sarcoplasmic reticulum works efficiently as long as a high local ATP/ADP ratio is maintained by the action of Cr kinase [116]. In the H_2_O_2_ cytotoxicity setting described above, for example, this might ameliorate the handling of intracellular calcium [165] and help the preserving of the mitochondrial calcium signals, thus favouring myoblast differentiation even in the course of oxidative stress [117].

Cr protects mtDNA from the oxidative damage caused by H_2_O_2_ in HUVECs [130] or repeated UV irradiation in human fibroblasts [131], through mechanisms involving both AO effects and normalization of cells' energy status. These effects might be responsible for the Cr-associated prevention of mtDNA loss caused by H_2_O_2_ in differentiating myoblasts [117]. Notably, the accumulation of mtDNA mutations is among the causative factors of sarcopenia [166].

Moreover, Cr stabilizes and prevents the opening of the mitochondrial permeability transition pore complex caused by a variety of adverse conditions, thus decreasing mitochondrial apoptotic susceptibility and cell death [167].

Hence, Cr ameliorates cellular energy status and at the same time protects the sites of energy production, that is, mitochondria, from ROS which would otherwise cause severe energy failure and cell death [167].

The last but not secondary distinctive feature of Cr as compared to generic AOs is its anabolic activity on skeletal muscle. This property has been documented since the ‘70s of the past century and has been demonstrated in a variety of *in vitro* [120, 168, 169] as well as ex vivo animal studies [168] or human studies [170–173]. Human studies involved training healthy adults [171], rehabilitating adults [170] or sedentary adults [172], and training [173] elderly. There is a general consensus on the fact that Cr-induced muscle anabolism depends mainly on its capacity to stimulate the mRNA expression of muscle regulatory factors, namely, Myo-D, Myf-5, MRF-4, and myogenin, and of IGF-1 [120] and to repress that of myostatin [169]. Other authors [174–177] reported positive effects of Cr supplementation on muscle mass and strength of exercising elderly and focused on the importance of the timing of Cr intakes, namely, pre- versus postexercise, to achieve better outcomes, postexercise intake being the most effective.

Given these premises, Cr could then represent a valuable dietary supplement to prevent muscle aging and sarcopenia. Interestingly, some of the effects elicited by supplementary Cr overlap with, and may reinforce, the positive ones induced by physical exercise (i.e., higher expression of PGC-1*α* and of muscle regulatory factors), and others (i.e., the ergogenic ones) do support physical training: on the whole, these peculiar traits are likely to give rise to a positive loop that, with regard to sarcopenia, suggests that supplementary Cr may positively interact with physical exercise. Indeed, recent studies on older adults and elderly people actually support this notion. Two distinct trials on community-dwelling older Canadian adults [178, 179] showed overall greater improvements in strength and higher gains in fat-free mass in Cr-supplemented participants, when compared with the placebo groups. A meta-analysis [180] of data from 357 older adults found enhanced benefits (in terms of lean mass, strength, and bone mass) of exercise training when combined with Cr supplementation. Another study showed that the addition of Cr enhances isometric strength and body composition improvements following strength exercise training in older adults [178]. A well-designed and more recent trial on 32 healthy, nonathletic men and women aged 60–80 years showed that three months of low-dose Cr supplementation associated with resistance training resulted in significant increases in lean mass [181]. In particular, unlike the placebo/resistance training group, the Cr/resistance training group (initial body, lean, android, and gynoid fat masses of 68.1, 38.3, 39.6, and 45.1 kg, resp.) showed a significant increase in muscle mass (+1.79 kg), a significant decrease in android and gynoid fat (−1.02 kg and −1.56 kg resp.), and a tendency to decrease body fat (−1.22 kg).

On the whole, the recognition of its pleiotropic nature, along with the encouraging outcomes of the trials on elderly people [127, 128, 173, 176, 178, 180–183], provides a strong rationale for considering and reappraising Cr as a valuable and safe supplement to counteract sarcopenia. As a final clue in support of this idea, it is worth noting that a study on mice demonstrated that Cr exerts a fair antiaging effect, since its chronic supplementation increases lifespan, improves health, and attenuates changes in biochemical and genetic markers associated with aging [184].

## 6. Coenzyme Q10 as Ergogenic and AO Mitochondrial Nutrient

CoQ is an endogenous isoprenylated quinone isolated for the first time in 1957 by Crane et al. from beef heart mitochondria [185]. Isoprenylation is responsible for the high lipophilic nature of this molecule that is ubiquitous in biological membranes, and therefore, it is also known as ubiquinone. The analysis of the literature shows that ubiquinone homologs are highly represented in various organisms from monera to vertebrates although they may differ in the number of isoprenoid units, in particular, ubiquinone in humans is present in the form of CoQ10, the homolog with ten isoprenoid units [186]. From a chemical point of view, CoQ10 is able to act as an electron and proton shuttle following two-electron redox reaction and reversible conversion from the oxidized form (ubiquinone) to the reduced form (ubiquinol). The redox properties of ubiquinone underlie its major biological functions, namely, to act both as an electron carrier intermediate in the mitochondrial respiratory chain and as a phenolic AO in the lipid environment. These two characteristics strongly link CoQ10 biochemistry to mitochondrial bioenergetics [187] and differentiate its role from other low molecular weight AOs. In fact, while CoQ is ubiquitous in all biological membranes (e.g., cellular organelles and plasma membrane) and in lipoproteins, it is of particular relevance to the inner mitochondrial membrane where the oxidative phosphorylation occurs and, consequently, to tissues characterized by high respiratory demand and energy turnover such as cardiac and skeletal muscle tissues [188].

Ubiquinol is a potent reducing agent that can interrupt the initiation of lipid peroxidation or also break the chain reactions by reacting with lipid peroxide radicals [189].

Ubiquinol acts mainly as a chain-breaking antioxidant in the membrane, reacting with carbon- and oxygen-centered radicals while its reactivity toward hydroperoxyl radicals is limited and much slower than that of tocopherol [190] one of the most potent exogenous antioxidants. Nonetheless, ubiquinol is more efficient against peroxidation of LDL (low-density lipoproteins) than *α*-tocopherol [191] that on a molar basis represents by far the major antioxidant in lipoproteins.

Ubiquinol and tocopherol interplay synergistically with important consequences in relation to the antioxidant activities of both molecules: ubiquinol regenerates tocopherol from oxidized tocopheryl radical, thus protecting the lipid environment from oxidation [188]; moreover, ubiquinol when compared with *α*-tocopherol has a higher reactivity toward both galvinoxyl and peroxyl radicals, since ubiquinol has two active hydrogens that react with oxygen radicals whereas *α*-tocopherol has only one. The reaction leads to the production of semiquinone radical which subsequently reacts with another radical X to give ubiquinone. In the case of peroxyl radicals, the steady state concentration of these radicals is usually low. Under these conditions, the life of semiquinone radical is longer and it has more chance to react with oxygen to give ubiquinone and hydroperoxyl radical. In this condition, hydroperoxyl radical may react with lipid to induce a lipid peroxidation, which results in the reduction of induction period or increase in the rate of lipid peroxidation. Under these conditions, ubiquinol does not act as an antioxidant [192].

However, in physiological conditions, tocopherol abundance in membranes scavenges hydroperoxyl radical and suppresses autoxidation of ubiquinol exerting a potent combined antioxidant effect [193, 194]. It is worth noting that, in mitochondria, the reduced form of CoQ is regenerated by the respiratory chain. In fact, both ubiquinol and the ubisemiquinone radicals can react with complex III and be suitably oxidized/reduced at the Qo and Qi sites of the enzyme. In addition, ubiquinone can be fully reduced by complex I, complex II, and other dehydrogenases of the inner mitochondrial membrane.

In the MRC, electrons donated from NAD- and FAD-dependent dehydrogenases funnel into CoQ as a common acceptor that in turn feeds into the cytochrome system [195, 196]. Growing evidence describes in detail the structure and function of the “core” respiratory complexes (i.e., complexes I, II, III, and IV) while several auxiliary enzymes have been also described to reduce ubiquinone [197] or to deliver electrons directly to oxygen (i.e., alternative ubiquinol oxidase) by bypassing complexes III and IV (see [198, 199]) of the respiratory enzymes [198]. Most certainly, ubiquinone/ubiquinol molecules bound to respiratory complexes coexist with a mobile pool of molecules in the lipid bilayer of the inner mitochondrial membrane. Notably, their physiological concentration is close to the Km (concentration yielding half-maximal velocity) of the respiratory enzyme CoQ [198]. This fact implies that even slight variations in the concentration of mitochondrial CoQ result in dramatic changes in the respiratory rate, further stressing the biological relevance of subliminal deficit in the biosynthesis and providing a rationale for the use of exogenous CoQ as an ergogenic supplement (see below for further discussion).

CoQ10 synthesis in eukaryotes comprises at least ten enzymatic reactions in large part along the mevalonate pathway where isoprenoid units are formed [200]. Although in physiological conditions the organism produces sufficient amount of CoQ10, a small proportion (3–5 mg/day) is also introduced through the diet, in particular, through meat and fish consumption [201]. Dietary CoQ10 is absorbed in the plasma, where it is vehiculated by lipoproteins and delivered to tissues. In pathological conditions, primary and secondary deficits in CoQ10 synthesis lead to severe mitochondrial impairment and are associated with different neurologic and muscular degenerative disorders [202–204]; moreover, pathological conditions associated with enhanced oxidative stress might result in an altered CoQ10 status [205]. Also, within physiological conditions, the CoQ10 status might be influenced in terms both of concentration and oxidative state by different factors including nutrition [201], physical activity [206], drug use [207–209], and aging [210–212]. In particular, in relation to the topic of this review, physical exercise is associated with a decreased CoQ10 plasma level, suggesting a higher tissue demand during training [206]. Moreover, CoQ10 synthesis in humans progressively declines after 20 years of age [210] as well as the activities of the reductases responsible for CoQ activation to its AO form, ubiquinol.

Accordingly, the abovementioned CoQ10 deprivation conditions are known to promote susceptibility of mitochondria to oxidative damage, as in the case of muscle toxicity caused by statins [207–209] commonly prescribed to elderly people. Notably, the entity of statins' adverse effects depends on multiple factors including genetic background [213, 214] and physical activity [215, 216] stressing the fact that even subliminal deficit in CoQ endogenous synthesis could significantly influence individual tolerance threshold. Taking into account that CoQ10 endogenous biosynthesis is also influenced by the mentioned extrinsic and intrinsic factors, notably, age and physical exercise, it is clear that muscle CoQ10 status deserves particular attention in the frame of age-related sarcopenia development.

According to the figures discussed so far, CoQ10 has been used over the last three decades as a nutritional supplement with ergogenic and AO activities to support mitochondrial bioenergetics and counteract oxidative stress in a wide range of clinical conditions [217–221].

However, the proven existence of respiratory SCs poses the question whether their role is compatible with the interpretation on bioenergetic grounds of the beneficial effect of orally administered exogenous CoQ10. In theory, by considering CoQ channeling within the SC, we might expect that ubiquinone content can be imposed by the amount of SC itself and therefore very much lower than the physiological CoQ10 concentration in the membrane. On the contrary, a careful reasoning based on the notion that bound CoQ within the SC is in chemical equilibrium with CoQ in the membrane pool supports the idea that even a slight decrease of endogenous ubiquinone content is sufficient to dissociate part of the quinone molecules from the SC. This consideration is also in line with the recently unveiled architecture of SC showing that the CoQ-binding sites of complex I and complex III are 10 nm apart and face a lipid microdomain that is open to the membrane [10]. In such a situation, it is likely that the function of the large amount of CoQ in natural mitochondrial membranes is to maintain the proper quinone compartmentation within the SC unit when it is formed: therefore, CoQ10 supplementation might also ameliorate a deficient respiratory activity.

Both bioenergetic and AO activities concur to optimize mitochondrial functionality and prevent oxidative damage that could fuel, through a vicious circle, mitochondrial ROS formation. Moreover, newly investigated action mechanisms of CoQ10, besides its free radical scavenging property, include the following: (1) a direct modulation of mitochondrial permeability transition pore (PTP); (2) anti-inflammatory activity; and (3) regulation of gene transcription. These mechanisms will be discussed in relation to their relevance to the physiology and biochemistry of the aging muscle and the potential role of CoQ10 in the prevention of sarcopenia.

PTP is a high-conductance protein channel located in the inner mitochondrial membrane [222]. In response to stress signals, mitochondrial depolarization causes the opening of the pore thus leading to increased permeability of the mitochondrial membranes to small molecules (<1500 daltons) in the intermembrane space, such as cytochrome c. The release of these factors in the cytoplasm is able to activate the process of cell death (apoptosis). PTP transition might be the result of acute injuries, such as hypoxia, insult in heart attack, or stroke, or might be the consequence of mitochondrial dysfunction leading to alterations of mitochondrial membrane potential and mobilization of Ca^2+^ stores.

While apoptotic pathways are required for normal cell turnover and tissue homeostasis, misregulation of programmed cell death is increasingly implicated in aging and aging-related diseases [223]. Indeed, in the aging muscle, apoptosis is known to be involved in muscle fiber atrophy and loss of myofibers, two common features of sarcopenia [224]. The onset of this degenerative process seems to stem from the accumulation of senescence-related mutations in mtDNA [130, 225–227], the concomitant development of MRC abnormalities triggering apoptotic and, ultimately, necrotic events. Moreover, sporadic denervation occurring in the elderly may also exacerbate these deleterious events [228]. Quinones have been shown to exert a direct effect on PTP [229] through a common binding site rather than through redox reactions. Occupancy of this site can modulate the PTP open-closed transitions, possibly through secondary changes of the Ca^2+^-binding affinity for the pore [230]. Evidences that CoQ10 inhibits the opening of the pore besides reducing the concentration of superoxide anion were obtained using a model of amyloid/oxygen glucose deprivation-induced neuronal excitotoxicity [231], in amitriptyline toxicity [232] and in ischemia and reperfusion in the heart [233]. These reports do not provide an evidence of a direct modulation of the pore by CoQ10: thus, this effect could be simply mediated by its AO capacity.

Concerning the CoQ10 anti-inflammatory effect, the recent literature suggests that it acts through the inhibition of NF-*κ*B nuclear translocation, preventing low-grade inflammation characteristic of inflammaging [60, 234].

The aging cell is characterized by a chronic proinflammatory state where NF-*κ*B acts as a key regulator of genes that encode cytokines, cytokine receptors, and cell-adhesion molecules [235–239]. Interestingly, CoQ10 supplementation has been shown to influence the expression of proinflammatory NF-*κ*B and stress-related gene activation, by modulating the expression of miR-146a both in LPS-stimulated monocyte [239, 240] and in young and senescent endothelial cells.

Finally, CoQ10 has been shown to influence the expression of a wide set of genes [240–244]. Modulation of inflammation described above represents a clear example where CoQ10 is able to influence the nuclear translocation of NF-*κ*B. This is probably achieved through the direct AO effect of ubiquinol similarly to other AOs [245], although a direct modulation of regulatory miRNAs cannot be ruled out. Moreover, differently from other AOs, a controlled prooxidant activity at the level of the MRC might contribute to the release of potent inducers of gene expression, such as H_2_O_2_. In particular, Linnane et al. [243] hypothesized that global gene expression regulation induced by CoQ10 in skeletal muscle is indeed achieved via superoxide formation with H_2_O_2_ as a second messenger to the nucleus. In a randomized controlled clinical study, human test subjects about to undergo hip replacement were given 300 mg CoQ10/die or placebo for 25–30 days before surgery: microarray gene expression patterns and muscle fiber type profiles from *vastus lateralis* samples showed a consistent CoQ10-dependent regulation of 115 genes with 47 genes upregulated and 68 downregulated in the CoQ10-treated subjects [246]. Moreover, a significant difference in the number of mitochondria-rich type I fibers was observed between placebo and CoQ10 patients. CoQ10-treated samples were also characterized by a higher proportion of type II fibers. These fibers have fewer mitochondria per cell and are more glycolytic with respect to energy requirements; they are involved in fast contractions and their proportion decreases with age [22]. The same authors concluded that the patients receiving CoQ10 have an altered fiber type composition more reflective of younger muscle than the group receiving the placebo.

Coenzyme Q10, in light of the abovementioned properties and biological effects and besides its long standing use in different clinical fields, has attracted considerable interest as a safe food supplement in human nutrition as well as specialised sport nutrition. Clinical trials supporting this application include animal studies [247] where senescence-accelerated mice supplemented up to 10 months with ubiquinol showed improved exercise capacity, measured as time of run on a treadmill until exhaustion; moreover, CoQ10 supplementation was shown to lower exhaustive exercise-induced muscular injury in rats by enhancing stabilization of muscle cell membrane [248]. In humans, trials have been conducted both with the use of ubiquinone and more recently with ubiquinol. In general, data have shown that supplementation in healthy trained individuals produces an increase in oxygen uptake, improvement in cardiac parameters, and enhanced resistance, as validated also by means of randomized crossover studies [249–251]. CoQ supplementation was also shown to be effective in reducing exercise-induced oxidative damage [221] and muscular injury in humans [221, 252].

## 7. Conclusions

The significant socioeconomic costs of musculoskeletal aging, in an expanding elderly population, have emphasized the need to develop effective, safe, and costless interventions to prevent and/or delay the progression of sarcopenia: to this aim, a likely candidate is the association of exercise training with AOs.

However, as we have extensively discussed, a profound reappraisal of the role of AOs in preventing sarcopenia is needed. Indeed, the agents categorized under the generic heading of “AO,” that is, capable of broadly and simply blocking ROS, have not provided until now clear evidence for their benefits. On the contrary, dietary AOs characterized by additive features, such as positive muscle tropism and multiple converging actions, might reveal a much more therapeutically profitable effect.

**Figure 1:**
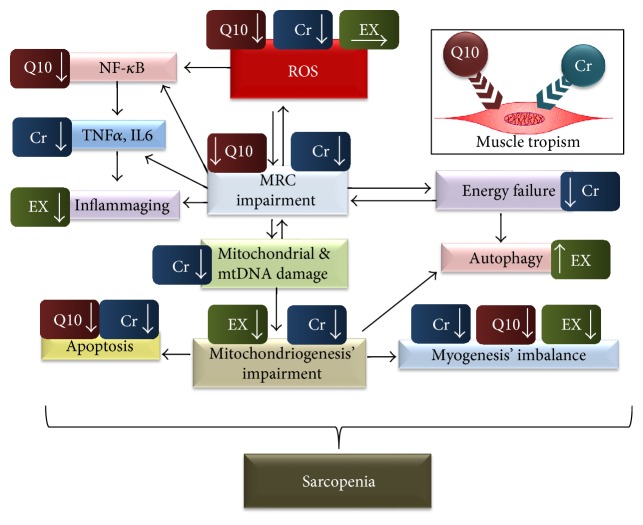
The role of ROS in the pathogenesis of sarcopenia and the multiple positive and converging effects promoted by Cr, CoQ10, or physical training. The sequences of relevant ROS-related pathogenetic events leading to sarcopenia are summarized in the flow chart, where the effects promoted by Cr, CoQ10, or physical exercise (corresponding to the labels “Cr,” “Q10,” and “EX” in the figure, resp.) are also illustrated. The types of the modulations caused by Cr, CoQ10, and EX are symbolized according to the following scheme: labels with the arrow pointing down = decrease, arrow pointing up = increase, and arrow pointing right = hormetic effect. The inset in the upper rightmost of the figure emphasizes the high accumulation of Cr and CoQ10 in skeletal muscles.

In this light, we believe that both Cr and CoQ10 can be considered as “muscle-specific pleiotropic AOs” capable of positively, if not synergistically, interacting with physical training to delay the onset and progression of sarcopenia (Figure 1). This topic requires further investigation, but it could pave the way for a better understanding of the mechanisms of skeletal muscle adaptation and plasticity and provide novel therapeutic targets to “reset” age-associated muscle loss.
